# The RNA-Binding Protein SBR (Dm NXF1) Is Required for the Constitution of Medulla Boundaries in *Drosophila melanogaster* Optic Lobes

**DOI:** 10.3390/cells10051144

**Published:** 2021-05-10

**Authors:** Ludmila Mamon, Anna Yakimova, Daria Kopytova, Elena Golubkova

**Affiliations:** 1 Department of Genetics and Biotechnology, Saint-Petersburg State University, Universitetskaya Emb. 7/9, 199034 St. Petersburg, Russia; mamon@LM2010.spb.edu or; 2 A. Tsyb Medical Radiological Research Center—Branch of the National Medical Research Radiological Center of the Ministry of Health of the Russian Federation, Koroleva Str. 4, 249036 Obninsk, Russia; anna.prosovskaya@gmail.com; 3 Institute of Gene Biology, Russian Academy of Sciences, Vavilov St. 34/5, 119334 Moscow, Russia; dvkopytova@gmail.com

**Keywords:** neurogenesis, optic lobe, *Drosophila*, NXF, RNA-binding protein

## Abstract

*Drosophila melanogaster sbr* (*small bristles*) is an orthologue of the *Nxf1* (*nuclear export factor 1*) genes in different *Opisthokonta.* The known function of *Nxf1* genes is the export of various mRNAs from the nucleus to the cytoplasm. The cytoplasmic localization of the SBR protein indicates that the nuclear export function is not the only function of this gene in *Drosophila.* RNA-binding protein SBR enriches the nucleus and cytoplasm of specific neurons and glial cells. In *sbr^12^* mutant males, the disturbance of medulla boundaries correlates with the defects of photoreceptor axons pathfinding, axon bundle individualization, and developmental neurodegeneration. RNA-binding protein SBR participates in processes allowing axons to reach and identify their targets.

## 1. Introduction

*Drosophila melanogaster sbr* (*small bristles*) is an orthologue of the *Nxf1* (*nuclear export factor 1*) genes in different organisms, including human [1,2,3]. The best known function of the *Nxf1* genes is the transport of various mRNAs from the nucleus to the cytoplasm [1]. NXF1 is localized in the nucleus or the nuclear envelope according to its function [4]. As we have shown previously, *D. melanogaster* SBR (Dm NXF1) is located not only in the nucleus but also in the cytoplasm of different cells [5,6,7]. This finding suggests that the SBR protein has specific cytoplasmic functions in addition to its participation in nuclear export of mRNAs. In the *Drosophila* larval brain, SBR forms granules in the neuron bodies and neurites [7]. Some granules contain two RNA-binding proteins, SBR and dFMR1 (drosophila Fragile Mental Retardation 1), which are known components of RNP-granules. Others are marked by either FMR1 or SBR. These observations suggest that SBR has specific localized RNA targets in the cytoplasm [8]. Regulated translation of localized mRNAs is a widespread and essential process during neurogenesis [9].

**Figure 1:**
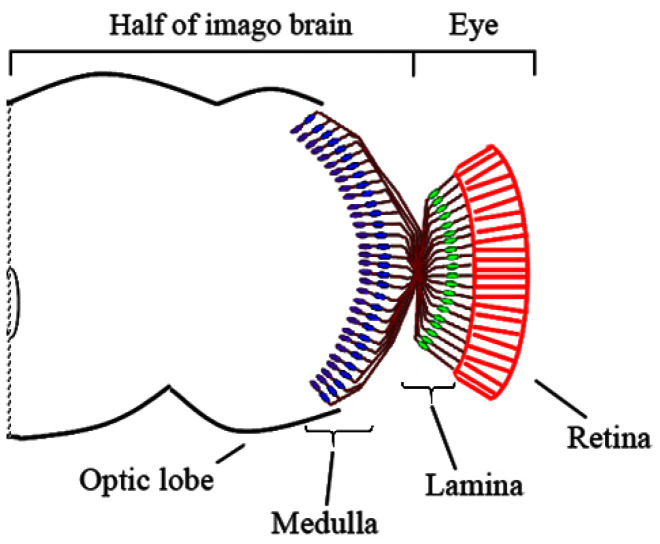
Location of the axon terminals of the R1–R8 photoreceptor neurons in an optic lobe of the imago brain. The R1–R6 axons terminate in the lamina (depicted in green), and the R8 (blue) and R7 (violet) axons terminate in the medulla (from [18] with changes).

Neurogenesis in *Drosophila* is a highly organized process that requires strict regulation of the compartments individualization and the establishment of the correct connections between neurons. Intercellular communications are an important mediator of axon guidance, proper axon bundle track, and neuron survival [10,11,12]. Together with RNA binding proteins, localized RNAs in complex create a system of rapid and long-term production of signal or receptor molecules near cellular membranes of glial cells, neurons and their neurites. This determines intercellular communications and allows for the appropriate boundaries to be established between the compartments in the brain. The optic lobes of the fly brain are a model of neuropil compartmentalization, playing a crucial role in the development of the nervous system. Each of the optic lobes is divided into four brain compartments (neuropils): lamina, medulla, lobula, and lobula plate [13]. The compound eyes of *D. melanogaster* consist of single units called ommatidia. Each ommatidium contains eight photoreceptor neurons (R1–R8) that project into the optic lobe of the brain following the neural superposition rule [14,15]. This rule ensures that the axons from the retina cells obtain information from the same point in space projected onto the same cartridge of the lamina or column of the medulla. The axons of the photoreceptor neurons R1–R6 form the lamina plexus in a retinotopic manner, subdividing the lamina neuropil into synaptic cartridges. Each cartridge contains terminals of the R1–R6 photoreceptor neurons located in the neighboring ommatidia and detect the same visual point of space [16]. The axons of the R7–R8 photoreceptor neurons cross the lamina and terminate in the medulla (Figure 1) [17,18].

Morphological defects in the optic lobes and ellipsoid body are the dominant features of *sbr^12^* mutant males, characterized by low activity in the negative geotaxis test [19]. We discuss the role of the *sbr (Dm nxf1)* gene in establishing medulla compartment boundaries in *Drosophila melanogaster* optic lobes. The defects of the photoreceptor axons pathfinding, axon bundle individualization, and developmental neurodegeneration are essential for the formation of medulla boundaries in *sbr^12^* mutant males. Non-random cytoplasmic localization of RNA-binding protein SBR suggests it plays a role in the processes allowing axons to reach and identify their targets.

## 2. Materials and Methods

### 2.1. Strains and Hybrids

The vitally important *D. melanogaster* gene *sbr* (*Dm nxf1*) is located on the X chromosome, whereas the orthologous *nxf1* genes of vertebrates are located on autosomal chromosomes. Since homozygous or hemizygous carriage of the *sbr^12^* allele is lethal, viable male carriers of *sbr^12^* can be obtained by crossing *sbr^12^/FM6* females with males that carry *sbr^+^* as a part of the *Dp(1;Y)y^+^v^+^* duplication on the Y chromosome. *sbr^12^/Dp(1;Y)y^+^v^+^* males differ from wild type males not only in the presence of the mutant *sbr^12^* allele, they also have two copies of the *sbr* gene. In *sbr^12^/Dp(1;Y)y^+^v^+^* males, the extra *sbr^+^* allele lies on the Y chromosome. This fact is especially important for genes normally localized on the X chromosome. The transcriptional level of X chromosome-linked genes is regulated by the dosage compensation mechanism [20]. Therefore, additional control variants are required to study *sbr^12^* phenotypes. *L4/Dp(1;Y)y^+^v^+^* males with a deletion of the *sbr* gene on the X chromosome (*Df(1)v-L4, ras^2^ m^D^*) and an *sbr^+^* allele on the Y chromosome provide an opportunity to explore the impacts of one copy of the *sbr^+^* allele due to its unusual localization.

Another additional control is *sbr^+^/Dp(1;Y)y^+^v^+^* males, which have a double dose of *sbr^+^* (one on the X chromosome and another on the Y chromosome). This control equalizes *sbr^12^/Dp(1;Y)y^+^v^+^* animals to *sbr^+^/Dp(1;Y)y^+^v^+^* animals in relation to the *sbr* gene dose. The additional controls help to differentiate the phenotypes of the *sbr^12^* allele from those of the double dose of the *sbr* gene and the unusual chromosomal localization of the *sbr^+^* allele.

Heterozygous males *sbr^12^/Dp(1;Y)* have full-size SBR protein along with the SBR^12^ protein that carried deletion of 10 a.a. (TIFITNATHE) at the C-terminal part of the protein. The *sbr^12^* allele has the dominant-negative effect on male fertility, locomotion activity, and the brain abnormality, what is characterizing the gain of function alleles. We considered the possibility of other mutations in the same X-chromosome and used recombination mapping for finding an additional mutant allele. Results are presented in Appendix A.

### 2.2. Sample Collection

Third instar male larvae were used to analyze the structure of the medulla in the developing brain, as well as to study the location of the photoreceptor neurons in the eye-antennal imaginal discs (EAIDs). EAIDs were dissected in a cold PBS solution. In this case, brains with intact EAIDs were dissected for immunohistochemical staining (see below for details).

The heads of 3–5-day-old adult males were dissected in cold PBS to examine the brain structure and the location of the photoreceptor neuron terminals in the medulla using fluorescent staining (see below for details). All brain centers are already formed at this age [21]. Paraffin sections of the imago head were stained with haematoxylin and eosin to analyze the optic lobe structure.

### 2.3. Immunohistochemical and Fluorescent Staining

Fixation of dissected larval and imaginal brains was performed in 4% formaldehyde in PBS for 15 min at room temperature. Each brain was washed 3 times for 15 min each in PBST (PBS supplemented with 0.1% Tween-20). Permeabilization was performed in PBS with 0.3% Tween-20 for 30 min at room temperature. Subsequently, the brains were incubated for 2 h in blocking solution (10% fetal calf serum (FCS) in PBST) at 4 °C to avoid non-specific antibody binding. Antibodies to horseradish peroxidase (HRP), conjugated with GFP (Jackson ImmunoResearch Laboratories, West Baltimore Pike, PA, USA, 1:250 in blocking solution, overnight at +4 °C) were used to identify the neuropils, the photoreceptor neurons, and their processes in the EAID and larval brain [22]. Rabbit polyclonal antibodies to SBR [23] were used for the visualization of SBR protein in *Drosophila* brain and EAID (1:100 in blocking solution, overnight at +4 °C). Mouse monoclonal antibodies to Chaoptin (24b10, Developmental Studies Hybridoma Bank, Iowa City, IA, USA) were used for the visualization of photoreceptor neurons and their processes in the imago brain (1:50 in blocking solution, overnight at +4 °C). Subsequently, each brain was washed 4 times for 15 min each in PBST and were stained with the secondary goat anti-rabbit antibodies, conjugated with AlexaFluor-647 (1:1000 in blocking solution, overnight at +4 °C) and/or secondary goat anti-mouse antibodies, conjugated with AlexaFluor-555 (1:1000 in blocking solution, overnight at +4 °C). Subsequently, each brain was washed 4 times for 15 min each in PBST and 2 times for 15 min each in PBS. Nuclei were stained with DAPI (1 µg/mL in PBS) for 15 min at room temperature. Each brain was then washed 3 times for 10 min each in PBS and mounted in Vectashield Mounting Medium (Vector Laboratories, Burlingame, CA, USA).

### 2.4. Head Paraffin Sections

Flies were fixed in 4% formaldehyde in PBS overnight at +4 °C. Dehydration and paraffinization was carried out using the standard method [24]. Serial head sections with a thickness of 5 μm were obtained using a microtome and transferred to glass slides coated with poly-l-lysine. Subsequently, the sections were deparaffinized, hydrated, and stained with haematoxylin-eosin using the standard method [24]. After that, the sections were dehydrated, incubated in xylene (2 times for 3 min), and mounted in Canadian Balsam.

### 2.5. Microscopy

The analysis of the preparations was carried out using a Leica TCS SP5 laser scanning confocal microscope (Leica Microsystems GmbH, Wetzlar, Germany) at the Chromas Center for Collective Use at the St. Petersburg State University and a Leica DMI4000 laser scanning confocal microscope based in the Radiation Biochemistry Laboratory of the A. Tsyb Medical Radiological Research Center, a branch of the National Medical Research Radiological Center of the Ministry of Health of the Russian Federation (A. Tsyb MRRC), Obninsk, Russia.

To visualize of the photoreceptors in the imago brain, autofluorescence was detected by means of excitation with a 488 nm exciting laser and a 530–580 nm blocking filter. With these scanning parameters, the autofluorescence of the photoreceptors with their axons is clearly observable, despite the presence of background autofluorescence from other tissues of *D. melanogaster*.

To visualize the internal structure of the lamina and medulla in the imago brain, autofluorescence of stained head paraffin sections was detected a laser with an excitation peak at 488 nm exciting laser and a 530–600 nm blocking filter. This approach made it possible to precisely focus on the surface of the paraffin section and to get a high-resolution image.

Fluorescence of anti-IgG::AlexaFluor-647, anti-HRP::GFP, and DAPI were detected following the manufacture’s manual.

The obtained images were processed using computer image analysis systems, such as LAS AF Lite.

V1.7.0 (Leica Microsystems GmbH, Wetzlar, Germany) and ImageJ, as well as Adobe Photoshop V7.0 (Adobe Systems Incorporated, San Jose, CA, USA) were utilized to arrange composite images and drawing explanatory marks (arrows, contours) to simplify the visualization of the results.

## 3. Results

### 3.1. Medulla Malformations in sbr^12^/Dp(1;Y)y^+^v^+^ Males

The medulla neuropil is a highly ordered structure consisting of multiple synaptic layers. Projections of the R7–R8 photoreceptors terminate in different medullar layers (M3 and M6, respectively) and form strictly ordered synaptic connections with projections of the lamina and medulla neurons. Medulla columns are oriented perpendicular to the ten synaptic medulla layers [25].

**Figure 2:**
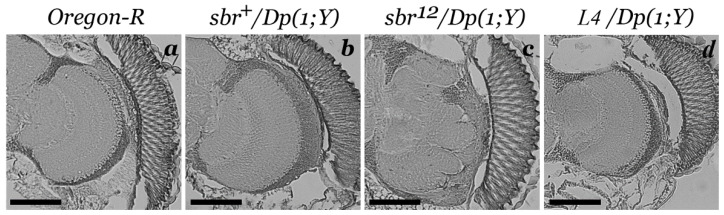
Paraffin sections of the optic lobes from the heads of males of different genotypes ((**a**)*—Oregon-R*, (**b**)*—sbr^+^/Dp(1;Y)y^+^v^+^*, (**c**)*—sbr^12^/Dp(1;Y)y^+^v^+^* and (**d**)*—Df(1)v-L4*, *ras^2^ m^D^/Dp(1;Y)y^+^v^+^*). Haematoxylin and eosin staining. The section at the level of the medulla is in the front third of the brain. Scale bar: 75 μm.

In *Oregon-R*, *sbr^+^*/*Dp(1;Y)y^+^v^+^* and *L4*/*Dp(1;Y)y^+^v^+^* adult males, the medulla has clear smooth boundaries (Figure 2a,b,d). Only in *sbr^12^/Dp(1;Y)y^+^v^+^* males, the medulla appears to have lost its integrity (Figure 2c). External boundary of the medulla in *sbr^12^/Dp(1;Y)y^+^v^+^* males looks like separate protrusions and hollows in all cases with variation of malformation degree. It is a dominant negative effect of the SBR^12^ protein rather than a consequence of the decrease in the content of the SBR protein, since similar disorders are absent in males *L4*/*Dp(1;Y)y^+^v^+^* with the *sbr* gene deletion (*L4*—*Df(1)v-L4, ras^2^ m^D^*) (Figure 2d).

**Figure 3:**
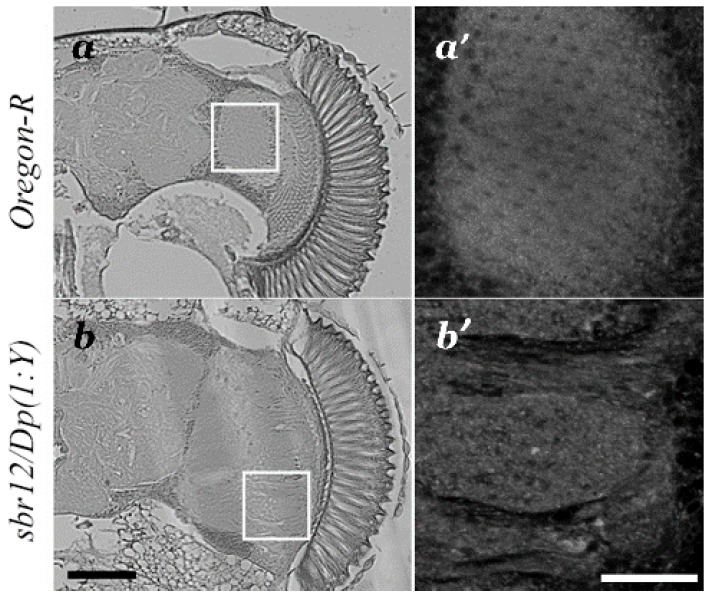
Paraffin sections of the adult *Drosophila* brain of *Oregon-R* (**a**,**a′**) and *sbr^12^/Dp(1;Y)y^+^v^+^* (**b**,**b′**) males. Architecture of medulla columns (**a′**,**b′**) are visualized by autofluorescence in the regions marked on the images (**a**,**b**). Medulla columns are ordered in *Oregon-R* males (**a′**) and are disorganized in *sbr^12^/Dp(1;Y)y^+^v^+^* males (**b′**). Scale bar: (**a**,**b**)—75 μm; (**a′**,**b′**)—25 μm.

The structure of the medulla columns was visualized by autofluorescence of adult *Drosophila* brain on the paraffin sections (Figure 3a,b). In wild type *Oregon-R* males, organization of the medulla columns is ordered (Figure 3a′). A striking feature of *sbr^12^/Dp(1;Y)y^+^v^+^* males is that the medulla columns are disorganized and non-structured (Figure 3b′).

**Figure 4:**
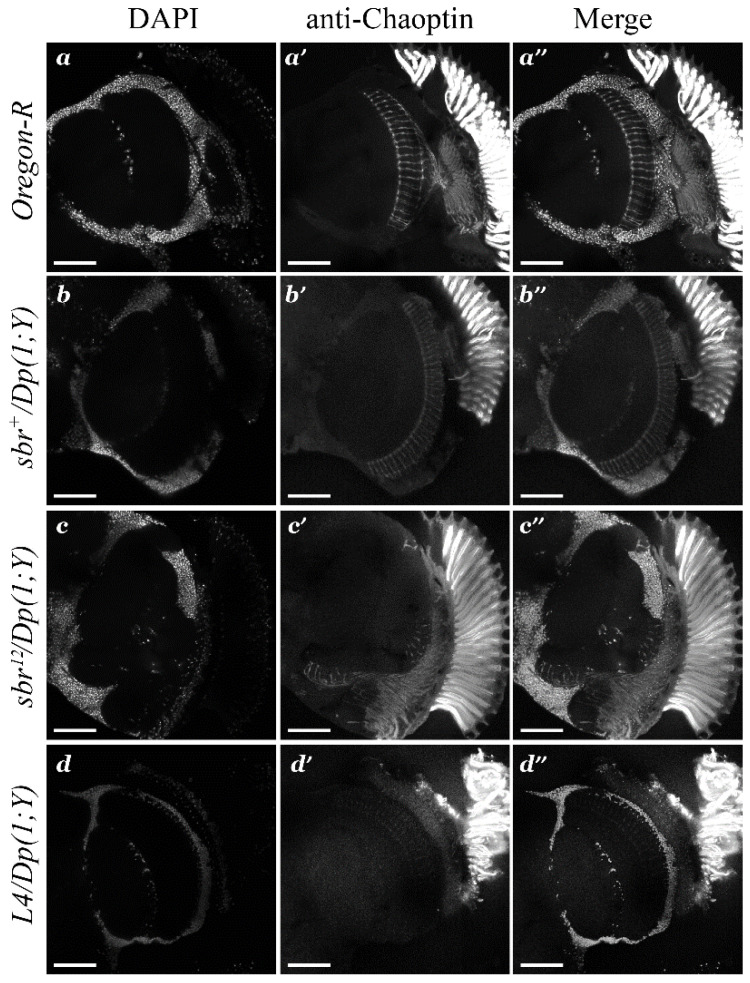
Axon terminals of the photoreceptors in the medulla of the optic lobe in adult males of different genotypes (**a**,**a′**,**a″**—*Oregon-R*; **b**,**b′**,**b″**—*sbr^+^/Dp(1;Y)y^+^v^+^*; **c**,**c′,c″**—*sbr^12^/Dp(1;Y)y^+^v^+^*; **d**,**d′**,**d″**—*Df(1)v-L4, ras^2^ m^D^/Dp(1;Y)y^+^v^+^*). Cell nuclei were stained with DAPI (**a**–**d**). Antibodies to Chaoptin allows to detect photoreceptor axon terminals (**a′**–**d′** and **a″**–**d″***—*merge). In *sbr^12^/Dp(1;Y)y^+^v^+^* axon terminals are not ordered and the medulla structure is disrupted (**c′**,**c″**). Scale bar: 50 μm.

The pattern of the structural disorganization of the medulla suggests that this disorganization could be caused by pathfinding defects in the photoreceptor axons. If that indeed were the case, then the axons of the photoreceptor neurons would not reach their targets and would not establish the correct connectome. To test this hypothesis, we investigated the locations of the photoreceptor terminals in the medulla of the whole adult *Drosophila* brain via confocal microscopy with the use of the antibody (Figure 4) and analysis of autofluorescence (Figure S1). The anti-chaoptin immunolabeling allows to visualize the terminal locations of the photoreceptors. The detection of the chaoptin demonstrated that the photoreceptor terminals were arranged in order, forming rows in the distal part of the medulla in the wild type (Figure 4a′,a′’), *sbr^+^*/*Dp(1;Y)y^+^v^+^* (Figure 4b′,b′’), and *L4*/*Dp(1;Y)y^+^v^+^* males (Figure 4d’,d’’). In *sbr^12^*/*Dp(1;Y)y^+^v^+^* males, the photoreceptor terminals were located chaotically and did not form ordered rows (Figure 4c′,c′’).

The R7 and R8 neurons are the pioneer neurons during the organization of the medullar layers. The axons of these photoreceptors grow first to the medulla and then serve as a navigational guide for the axons of other neurons, which participate in the formation of the individual columns [26]. Therefore, the breach of the boundary between the lamina and the medulla in *sbr^12^*/*Dp(1;Y)y^+^v^+^* males suggests that the process of coordinated termination of photoreceptor neurons is defective (Figure 4c′,c′’). These types of disruptions may indicate both problems with pathfinding of the axons of photoreceptor neurons to their targets, and with the specification of photoreceptor neurons. To shed light on the reasons for the observed defects, we investigated the formation of the photoreceptor axons at earlier developmental stages.

### 3.2. Defects in Fasciculation of Photoreceptor Axons in Eye-Antennal Imaginal Discs of sbr^12^/Dp(1;Y)y^+^v^+^ Male Larvae

Antibodies to the plant glycoprotein horseradish peroxidase (HRP) recognize the fly neuronal membrane [22] and allow for the identification of photoreceptors with their neurites. In the eye part of the eye-antennal imaginal discs (EAIDs) of the *Drosophila* third instar larvae, the projections of photoreceptors belonging to different ommatidia interact with each other via cell adhesion proteins. They form axonal tracts that then further consolidate into the optic nerves that connect each EAID to the respective optic lobe of the developing brain.

**Figure 5:**
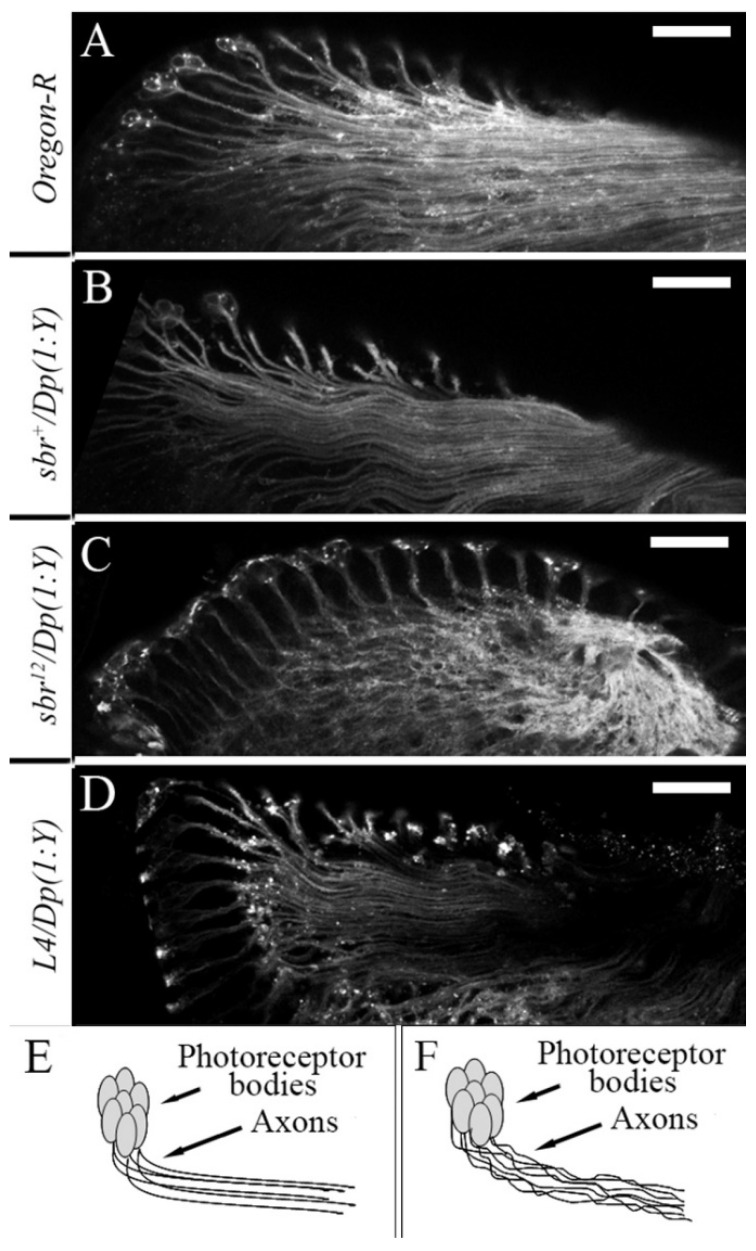
Localization patterns of the photoreceptor axons in the EAIDs of third instar male larvae. The photoreceptors and their axons were labelled with antibodies against HRP. (**A**–**D**)—maximal projections of the optic section series through the EAID zone, which contains photoreceptors and their axons, in males with the following genotypes: (**A**)*—Oregon-R*, (**B**)*—sbr^+^/Dp(1;Y)y^+^v^+^*, (**C**)*—sbr^12^/Dp(1;Y)y^+^v^+^*, and (**D**)*—Df(1)v-L4, ras^2^ m^D^/Dp(1;Y)y^+^v^+^*. Schematic images of photoreceptor axon localization: (**E**)*—Oregon-R*, (**F**)*—sbr^12^/Dp(1;Y)y^+^v^+^*. In *sbr^12^/Dp(1;Y)y^+^v^+^* males, the axon bundles are weak ordered and do not form parallel rows (**C**,**E**); the photoreceptor axons are parallel to each other in males of control genotypes (**A**,**B**,**D**,**E**). Scale bar: 20 μm.

Only *sbr^12^/Dp(1;Y)y^+^v^+^* males have a defective fasciculation (bundling) of the axons of the photoreceptor neurons in the EAIDs (Figure 5C). The axon bundles were located chaotically (Figure 5C,F) and not in parallel rows as observed in males of control genotypes (Figure 5A,B,D,E).

This is consistent with disorganized structure of the medullar columns in *sbr^12^/Dp(1;Y)y^+^v^+^* males (Figure 3b′). The defects in the structure of the photoreceptor axons observed in the EAIDs of *sbr^12^/Dp(1;Y)y^+^v^+^* males suggested that the *sbr^12^* allele affects axon bundle organization. The axons of the photoreceptor neurons form the optic nerve passing from the EAIDs to the brain. The most impressive phenotype of the *sbr^12^* allele is the disturbance of medulla boundary and column architecture in the adult *Drosophila* brain (Figure 2c and Figure 3b′). These data suggest that the *sbr^12^* allele affects multiple processes, including axon growth, arborization, establishment of new cell-cell interactions, and the formation of the strictly ordered zone of the R axons.

### 3.3. Features of Neurodegeneration in the Medulla of sbr^12^/Dp(1;Y)y^+^v^+^ Third Instar Male Larvae

**Figure 6:**
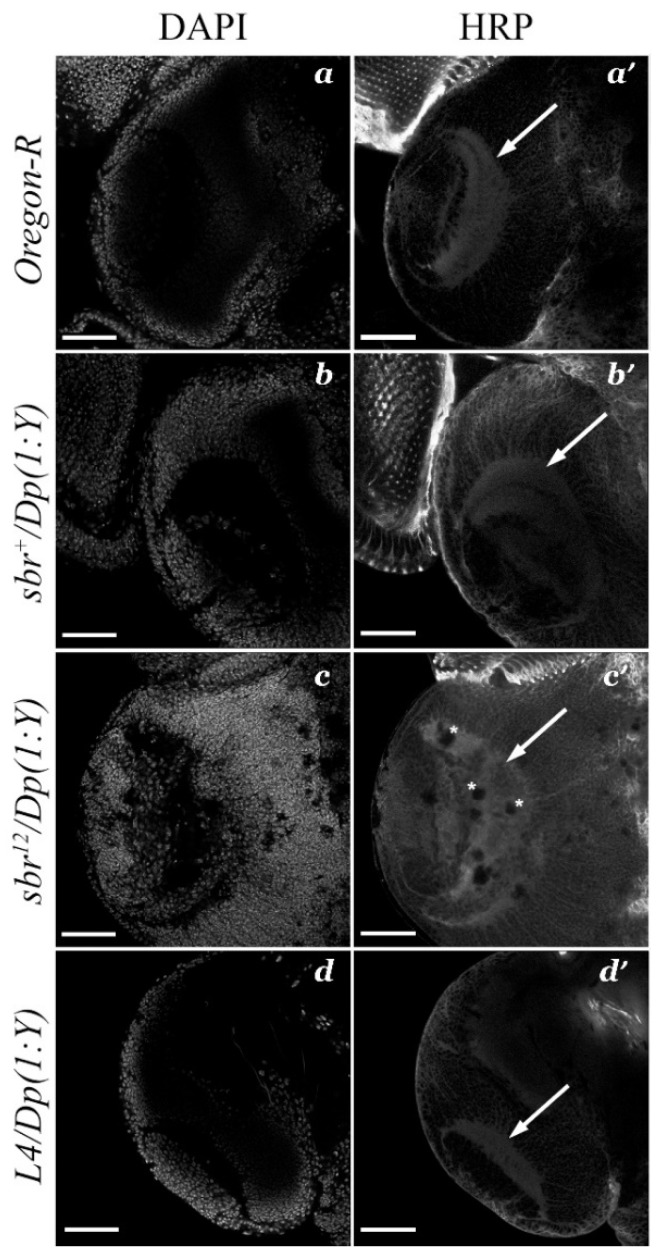
The medulla structure of third instar male larvae with different genotypes (**a**,**a′**—*Oregon-R*, **b**,**b′**—*sbr^+^/Dp(1;Y)y^+^v^+^*, **c**,**c′**—*sbr^12^/Dp(1;Y)y^+^v^+^* and **d**,**d’**—*Df(1)v-L4, ras^2^ m^D^/Dp(1;Y)y^+^v^+^*). Defects in the medulla (arrows) structure, disturbances in the nuclei localization pattern and manifestations of neurodegeneration (round zones with neither DAPI nor anti-HRP staining, with examples indicated by asterisks) were detected only in *sbr^12^/Dp(1;Y)y^+^v^+^* males (**c′**). Cell nuclei were stained with DAPI (**a**–**d**), and the neuropil was visualized with antibodies against HRP (**a′**–**d′**). Scale bar: 50 μm.

Staining with antibodies against HPR allows for the visualization of neuropils in the *D. melanogaster* larval brain. An analysis of the medulla structure in the developing optic lobes revealed that only *sbr^12^/Dp(1;Y)y^+^v^+^* males have features of neurodegeneration, including round black spots without any visual markers (Figure 6c′). Moreover, *sbr^12^/Dp(1;Y)y^+^v^+^* males have a defective medulla shape and localization pattern of the neuron bodies in this zone of the brain. The medulla neuropil normally looks like a sickle during the third instar larvae. This zone was easily visualized with antibodies against HRP (Figure 6a′–d′), and the cell nuclei were distributed on the periphery of the medulla neuropil (Figure 6a–d). In *sbr^12^/Dp(1;Y)y^+^v^+^* males, the medulla had a rough edge and a disordered structure. This abnormal medulla structure suggests that neuropil morphogenesis is defective in *sbr^12^/Dp(1;Y)y^+^v^+^* males (Figure 6c,c′). Males of control genotypes did not show signs of neurodegeneration in the medulla or defects in neuropil structure (Figure 6a′,b′,d′).

Neurodegeneration in a developing medulla of the *sbr^12^/Dp(1;Y)y^+^v^+^* males supports our assumption that the disruption of the structure of synaptic columns and cartridges in the medulla and lamina could be caused by defects in photoreceptor axons targeting, because mistargeted neurons and their axons are eliminate during brain development [27].

### 3.4. Localization of SBR Protein in Different Brain Cells

**Figure 7:**
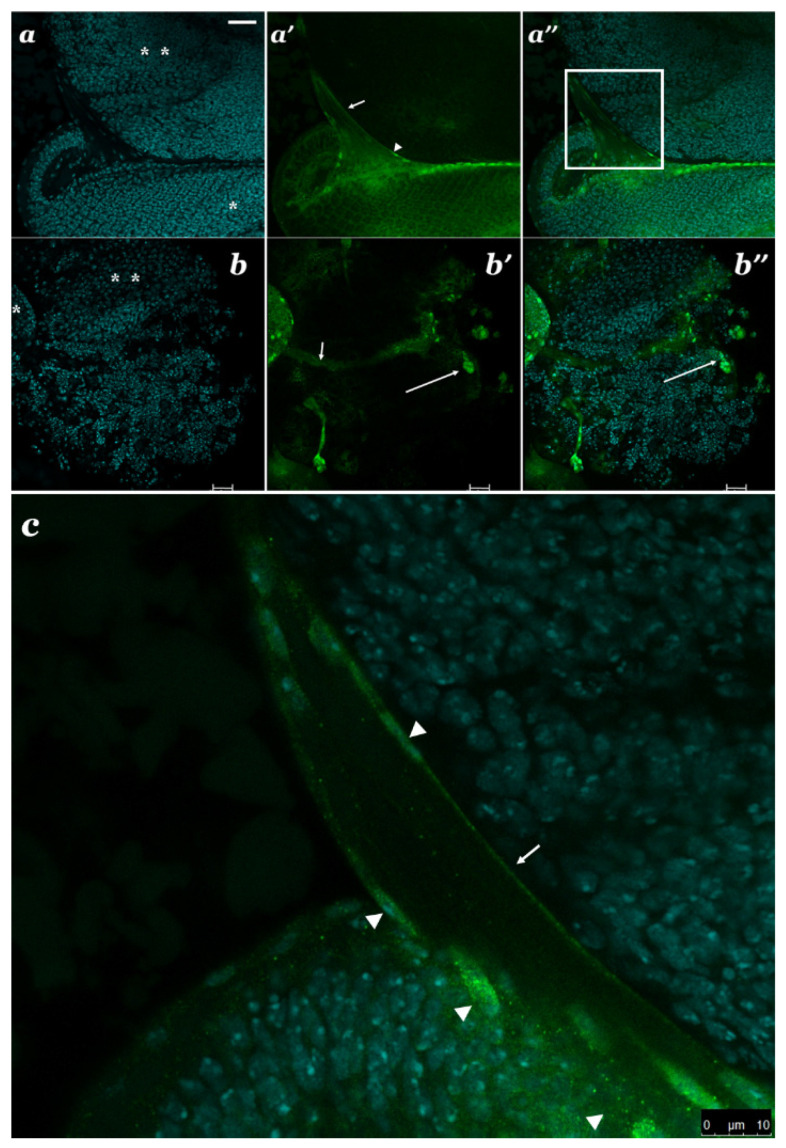
The optic section through optic stalk (arrow) connecting eye-antennal imaginal disc (EAID) (*) with the brain (**) of third instar larvae (*Oregon-R*). Localization of SBR in cells of the brain and/or EAID: DAPI for DNA in nuclei (blue) (**a**,**b**); anti-SBR antibody (green) (**a′**,**b′**) and merge (**a″**,**b″**); (**c**)*—*magnified image of the fragment from the **a′’** (framed). SBR enriches photoreceptor axons and the cytoplasm and some nuclei (arrow head) of proposed glial cells (**a**′,**a″**,**c**). In EAID, the ommatidia boundaries is also detected by anti-SBR that looks like a green mesh (**a**′). Anti-SBR allows to detect not only optic stalk (arrow) (**a′**,**b′**), but also several neuroblasts (neuronal stem cells) (long arrow), and their daughter cells (**b**′,**b**″). SBR marks nuclei and the cytoplasm of these cognate cells. Scale bar: (**a**)*—*25 µm, (**b**)—20 µm, (**c**)—10 µm.

Distribution of SBR in the brain as a whole and in specific cells can help understand this gene’s functions by studying the results of the immunofluorescence analysis. Antibodies to the N-terminal end (2–112 aa) of SBR [23] are used to detect the distribution of SBR in different cells. SBR enriches the nuclei and the cytoplasm of some cells: the glia and the axons of photoreceptor neurons in the optic stalk (Figure 7a,a′,a″,c), and some of the stem cells, which are the largest cells in the brain, and their progenies (Figure 7b,b′,b″). SBR is localized both in the nuclei and the cytoplasm of these cells.

Without special markers to glial cells it is not possible to detect cell specificity only by the morphological criteria alone. SBR enriches the cytoplasm and some nuclei of the glial cells ensheathing the optic stalk (Figure 7a′,a′’,c). Glial functions are required for the proper development and survival of neurons, and a defective glia becomes the reason for multiple developmental abnormalities, including neuronal degeneration [12]. The glia plays an important role in establishing the boundaries between visual centers of the brain [28]. This could be an argument in favor of the participation of SBR participation in processes allowing axons to reach and identify their targets.

## 4. Discussion

### 4.1. The Assumed Cytoplasmic Functions of the SBR RNA-Binding Protein, an Orthologue of the Evolutionarily Conserved NXF1 Protein

The SBR (NXF1) protein participates in RNA nuclear transport. However, it has no specificity for different mRNA targets. The interactions between NXF1 and mRNAs do not occur directly, as special adapter molecules are required [1,29].

The *sbr^12^* allele carries a 30-nucleotide deletion in exon 9 [30]. The deletion of 30 bp does not shift the reading frame in the coding part of the *sbr* gene. In SBR^12^ protein, 10 amino acids (TIFITNATHE) are absent. As a result, the structure of the NTF2L domain is altered. The NTF2L domain is responsible for an interaction with the NXT1 (p15) protein, which is a permanent partner of NXF1 [4,31]. Moreover, the NTF2L domain (as well as the UBA domain) mediates the interaction between NXF1 and nucleoporins (the proteins of nuclear pore complexes) and subsequent transport of all RNP particles from the nucleus to the cytoplasm [31,32]. The structure of the NTF2L domain is important for interactions between NXF1 and various mRNAs and especially for its interaction with the intron-containing transcript of the *nxf1* gene, which was established in vertebrates [33,34]. It is unknown whether the analogous intron-containing transcript of the *sbr* gene is a specific target for *Drosophila* SBR in the cytoplasm. The intron-containing transcript is more abundant among the *sbr* transcripts in the fly’s head [35]. It is unknown whether the intron-containing transcript is the specific target of SBR in neurites.

The defects in the specific brain centers of *sbr^12^* male cannot be simply explained by a decrease in overall mRNA export. The *sbr^12^* allele has a dominant negative effect on the background of the normal *sbr^+^* allele. We cannot exclude the possibility of selective effects of the *sbr^12^* allele on the nuclear transport of some mRNA targets. The presence of SBR^12^ may affect the export activity of specific mRNAs, which might include localized mRNAs important for neurogenesis. SBR is founded in ribonucleoprotein complexes in neurites, not all neuronal RNP granules are marked by the SBR protein. This observation suggests that SBR can also bind to its specific target RNAs in the cytoplasm [7].

Directed growth of the neurites allows them to reach their targets, and arborization and the establishment and maintenance of connections between neurons depend on dynamic changes in the cytoskeleton with regulated localized translation of localized mRNAs [36]. Non-random distribution of SBR in the cytoplasm of various cells, including nervous system cells [7], suggests that localized mRNAs associated with the cytoskeleton may be among the targets of SBR [8].

In the eye imaginal discs of *sbr^12^/Dp(1;Y)y^+^v^+^* males, the axons of the photoreceptors from the different ommatidia did not form strictly ordered bundles. This observation suggests a disruption in the connections between these axons and their terminals and targets. We cannot exclude the dominant-negative influence of the *sbr^12^* allele on the nuclear export of specific mRNAs. We have shown nonrandom cytoplasmic localization of the SBR proteins, which suggest additional cytoplasmic functions of the *sbr* gene. SBR is often localized near the cellular membrane (Figure S2). In the cytoplasm of various cells, SBR is found involved in granules (Figure 7c) [7], which are likely to be RNP-granules, since SBR is an RNA-binding protein. RNP-granules are quite complex and may contain proteins of signaling systems such as kinases and phosphatases [37]. This is required for the regulation of a localized mRNAs translation. The history of the discovery of TAP/Hs NXF1 (human ortholog of SBR) should be considered. TAP was first described as a cell adhesion factor (TAP—**T**ip **A**ssociated **P**rotein, where tip*—***T**yrosine kinase **I**nteracting **P**rotein) [38]. Only later NXF1 (TAP) was identified as a protein involved in the nuclear export of different mRNAs [39].

Cell adhesion molecules play important roles not only in fasciculation but also in axon targeting, and their malfunctions lead to morphogenesis defects [40,41]. Furthermore, neurons or their processes that form incorrect connections are usually destroyed [42,43,44,45]. This process might explain the neurodegeneration observed in the medulla in the brain optic lobes in *sbr^12^/Dp(1;Y)y^+^v^+^* male larvae (Figure 6c,c′). Establishment of the correct contacts between neurons and their targets is a necessary requirement for neuron survival [27,42]. The structural defects and neurodegeneration foci in the medulla of *sbr^12^/Dp(1;Y)y^+^v^+^* adult males may also be consequences of the elimination of neurons that formed aberrant synaptic connections. It is important to note that the predominant location of the areas of neurodegeneration in the medulla was consistent with defects in the photoreceptor targeting in this neuropil. Since R-neurons assume the role of pioneer neurons in the formation of the medulla, the elimination of their axons can cause of the medulla malformations. There are many mechanisms of neurodegeneration, and defects in RNA-binding proteins have been proposed to be a cause of neurodegeneration [46,47,48,49]. The defects of axon pathfinding, photoreceptor axon bundle individualization, and neurodegeneration can occur due to malfunction of intercellular communications [12].

### 4.2. The Non-Random Distribution of SBR in the Different Brain Cells

The non-random distribution of SBR in the brain and in the eye-antennal imaginal disc (EAID) as a whole, and the characteristic subcellular localization of this protein, indicates the importance of SBR in the formation of certain structures during larval development. The SBR marks the optic stalk (Figure 7a′,a″,b′,b″,c) and some neuroblasts with their daughter cells (Figure 7b′,b″). The ommatidia boundaries are marked by anti-SBR in the eye-antennal imaginal disc that looks like a green mesh. All marked by SBR structures are involved in forming the medullar boundary facing the lamina. In the optic stalk, some glial cells and axons are enriched by SBR (7a′,c). The contacts of glial cells with neurons, including their axons, are required for glial cells migration [50]. Glial cell migration is regulated by the external signals causing changes in the cytoskeleton [51,52]. At the same time, glial cells themselves are the source of extracellular signals guiding the pathway of axons to their targets within the developing brain [53]. Axon pathfinding is exceptionally sensitive to the perturbation of the actin cytoskeleton [54,55]. The localization of SBR in the cytoplasm of glial cells and in the axons further supports the hypothesis that RNA-binding protein SBR is essential for cell–cell communications forming the boundaries between the brain compartments.

There are special markers that identify glial cells. A glia specific expression of the genes *reversed polarity* (*repo*) or *glial cells missing* (*gcm*) allows to distinguish glial cells from neurons [12]. Nevertheless, there are some types of glial cells which can be recognized by the morphological criteria. On these criteria, it can be concluded that the SBR protein is enriched the nuclei and the cytoplasm of glial cells connected with the optic stalk (Figure 7a′,a″,b′,b″,c). All photoreceptor axons come together in a thick bundle called an optic stalk, which connects the eye imaginal disc to the brain [56]. Various types of glial cells associate with the optic stalk [57,58]. The role of SBR in the functioning of specific types of glial cells is unknown.

Cytoplasmic localization of the SBR suggest the existence of the prospective RNA-targets in glial cells, as well as in neurons. This is particularly relevant for large specialized cells whose function is to rapidly respond to external signals. The wrapping glia is important for the spatial distribution of the axons of the photoreceptor neurons [59]. The glia is involved in the photoreceptor axonal projection [59]. In *sbr^12^/Dp(1;Y)y^+^v^+^* males, the defects of signaling between wrapping glia and axon bundles may result in the disordered axonal projections. Furthermore, the interaction between neurons and glia is essential for the establishment of sharp compartment boundaries in the brain [60,61].

### 4.3. Allele-Specific Phenotypes of *sbr* Gene Mutations

*sbr^12^/Dp(1;Y)y^+^v^+^* males are characterized by abnormalities in sexual behavior and lower activity in the test for negative geotaxis [19]. The sterile *sbr^12^* males do not have mobile spermatozoa [6]. Spermatozoa immobility is an allelespecific phenotype of *sbr^12^*, *sbr^17^*, and *sbr^18^* alleles. The male sterility of flies with mutant *sbr* alleles is accompanied by axonemal abnormalities (i.e., defects in the cytoskeleton structure). The *sbr^1^* and *sbr^2^* alleles cause defects in bristle morphology, but homo- and hemizygotes animals are viable [62]. The bristles are a sensory organ of *D. melanogaster* whose development is a classic example of the realization of positional information during cell differentiation. As a rule, after asymmetric division, two daughter cells have unequal distributions of cytoplasmic determinants (including RNP complexes) and differ in their spindle apparatus orientation [63,64,65,66]. For example, segregation of neuroblasts from neuroepithelial cells and the subsequent asymmetric neuroblast divisions also depend on spindle orientation and non-random distribution of cytoplasmic determinants, which provide positional information.

NXF1 is a component of different macromolecular complexes, and it interacts with proteins and RNAs [67]. The specificities of these interactions are determined by the domain structure of an NXF1 (it contains a LRR (leucine-rich repeat) domain that controls protein-protein interactions, an NTF2L (nuclear transport 2 like) domain that supports an interaction with its partner protein NXT1, and an RBD (RNA-binding domain) and by conformational features of the entire molecule [34,68].

Defects in one part of the NXF1 molecule may affect functions of some complexes without having significant effects on the functions of others. The propensity of NXF1 to multimerize imparts a wide range of variability in heterozygous individuals. RNP complexes can contain different ratios of normal to mutant protein subunits, and this variability can affect structural and functional features of the complex and explain variations in the dominant negative effects due to the presence of mutant subunits.

## 5. Conclusions

SBR protein is required for the formation of the inner structure and the establishment of boundaries in the medulla of the *Drosophila* visual system. Morphogenetic events and the establishment of brain compartment boundaries are influenced by the cytoskeleton and membrane-associated RNP-complexes. The disorganization of the brain compartment boundaries is also aggravated by the degeneration of neurons with abnormally terminated axons. The formation of brain compartment boundaries depends on intercellular communication with local translation participation. The distribution of SBR in the nucleus and cytoplasm of specific neurons and glial cells suggests specialized functions of this protein. Determining of the composition of cytoplasmic granules containing SBR and enriching specialized cells will undoubtedly contribute to the understanding of the functions of SBR in the cytoplasm.

## Supplementary Materials

The following are available online at https://www.mdpi.com/article/10.3390/cells10051144/s1, Figure S1: Axon terminals of the photoreceptors in the medulla of the optic lobe in adult males of different genotypes (a,a′—Oregon-R, b,b′,c,c′—*sbr^12^/Dp(1;Y)y^+^v^+^*). Cell nuclei were stained with DAPI (a–c). Autofluorescence allows to detect photoreceptor axon terminals (a′,c′). Arrows indicate the R7–R8 axon terminals in the distal part of the medulla in Oregon-R (a′). In *sbr^12^/Dp(1;Y)y^+^v^+^,* autofluorescence (b′,c′) is weaker, then in *Oregon-R*. The medulla structure is disrupted (b,b′). Axon terminals are not ordered and can be visualized by autofluorescence in the region marked on the top image (b) with higher magnification (c′). Scale bar: a, a′,b,b′—50 μm; c,c′—25 μm, Figure S2: Localization of the SBR protein in cells of the salivary glands of *Drosophila*. The SBR protein is mark nuclear envelop and cellular membrane. (a) DAPI (DNA stain); (b) phalloidin (F-actin stain); (c) anti-SBR; (d) merge. Scale bar: 50 μm.

## Appendix A

Recombination mapping of lethal mutations for confirm the single mutation presence on the x chromosome in *sbr^12^*/*Dp(1;Y)y^+^v^+^* males.
