# Supplementary material for: The RNA-Binding Protein SBR (Dm NXF1) Is Required for the Constitution of Medulla Boundaries in Drosophila melanogaster Optic Lobes
Source: Cells. 2021 May 10;10(5):1144. doi: 10.3390/cells10051144 (PMC8151460; doi:10.3390/cells10051144)

We considered the possibility of some other mutation carried on the same chromosome as soon as identify first dominant phenotype of the $sbr^{12}$ allele. It is the male sterility. $sbr^{12}/Dp(1;\ Y)y^{+}v^{+}$ males do not copulate with virgin females. Other feature of such males is the locomotion defects. We tried to divide the lethality ($sbr^{12}$ is lethal allele, as majority of other *sbr* alleles) and male sterility by recombination. We used *ras v* markers for it. Genetic distance between *ras* and *v* is 1 cM. All recombinant males ($ras\ v^{+}$ or $ras^{+}\ v$) among the offspring of the $ras\ sbr^{+}\ v/ +\ sbr^{12}\ +$ females were fertile and have no behavior defects. Additionally, there are other alleles of the gene *sbr* with allele-specific male sterility. It is the argument for that $sbr^{12}$ may be allele with dominant male sterility.

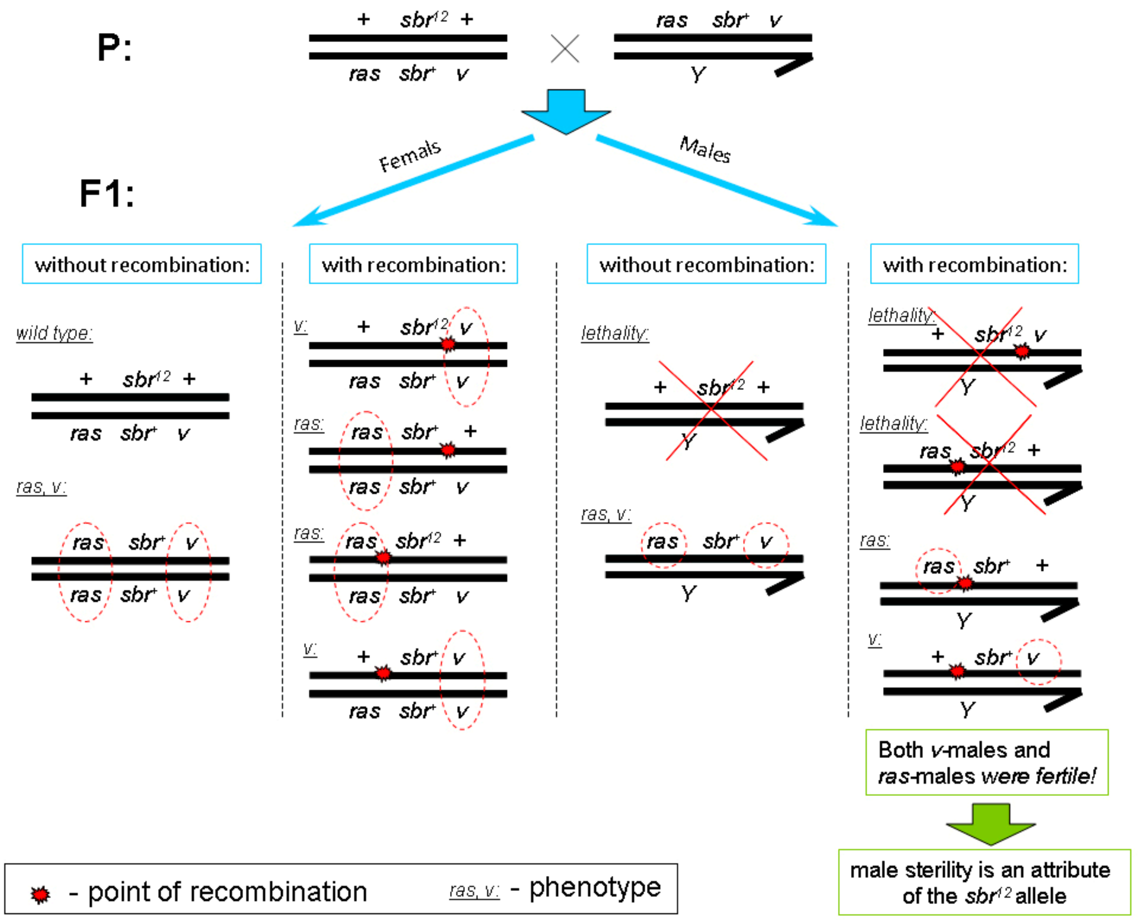

We have also considered another possibility: it can be additional lethal on the same chromosome with dominant effect on locomotion activity, and its lethal effect is compensated by *Dp(1;Y)y+v+*. We tested this assumption. We mapped the lethal mutations by recombination. The *sbr*[12]*/FM6* females were crossed with the *y cv v f car* males, then the hybrid females F1 were crossed with the *y cv v f car* males. All recombinant males were tested on locomotion activity to recessiveness verify. They were normal on their locomotion activity.

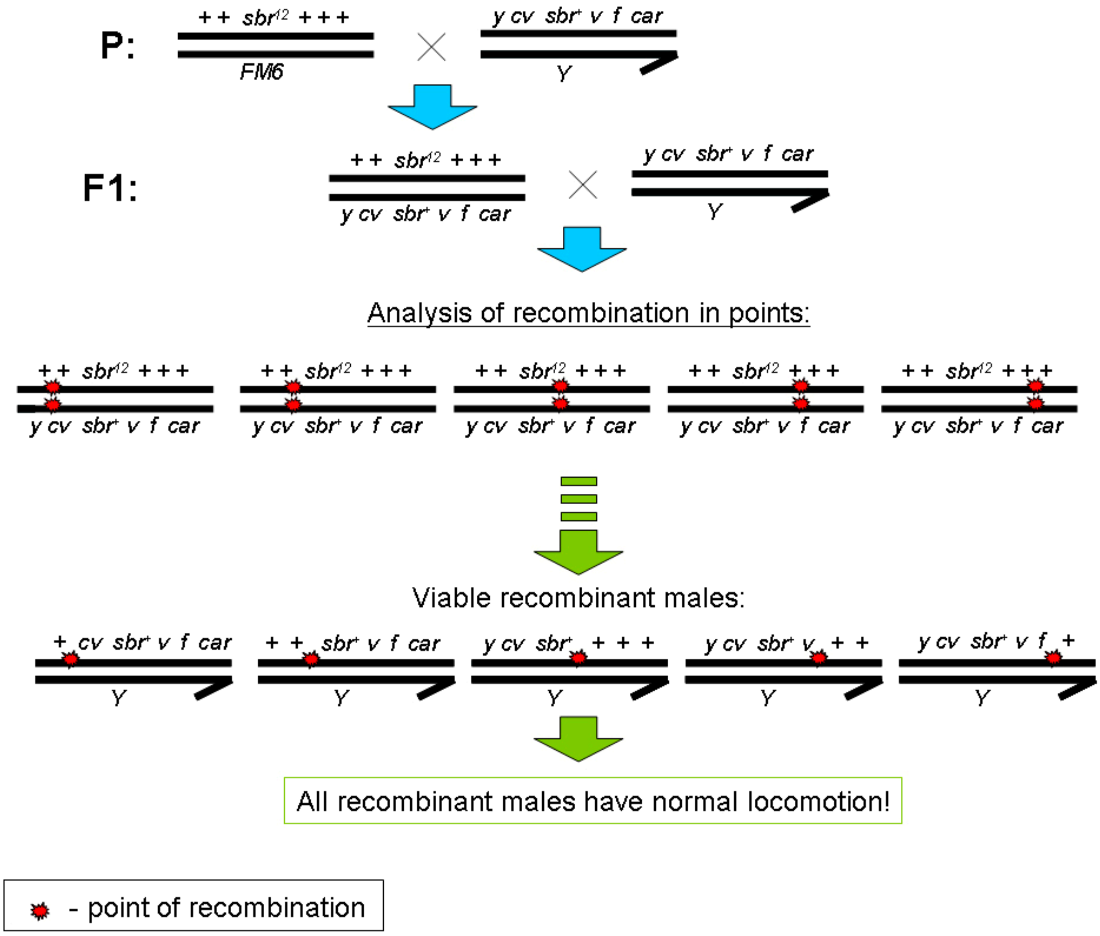

The mapping of the lethal mutations gives a possibility to answer the question: whether two lethal alleles of the different genes present on the tested X-chromosome. The analysis of phenotypic classes among male progeny allows to detect the frequency of recombination (segregation) between *cv* and $sbr^{12}$, and between $sbr^{12}$ and *f*. Sum of these frequencies was compared with the frequency of recombination between *cv* and *f* on results of cleavage among female progeny. Genetic distances between other markers were the same calculated for males and females. Genetic distance between all other markers were similar being calculated among male or female offspring in crossing the $sbr^{12}$/*y cv v f car* females with the *y cv v f car* males. The results conclude that $sbr^{12}$ is the only one lethal allele on X-chromosome of the $sbr^{12}/Dp(1;Y)y^{+}v^{+}$ males.

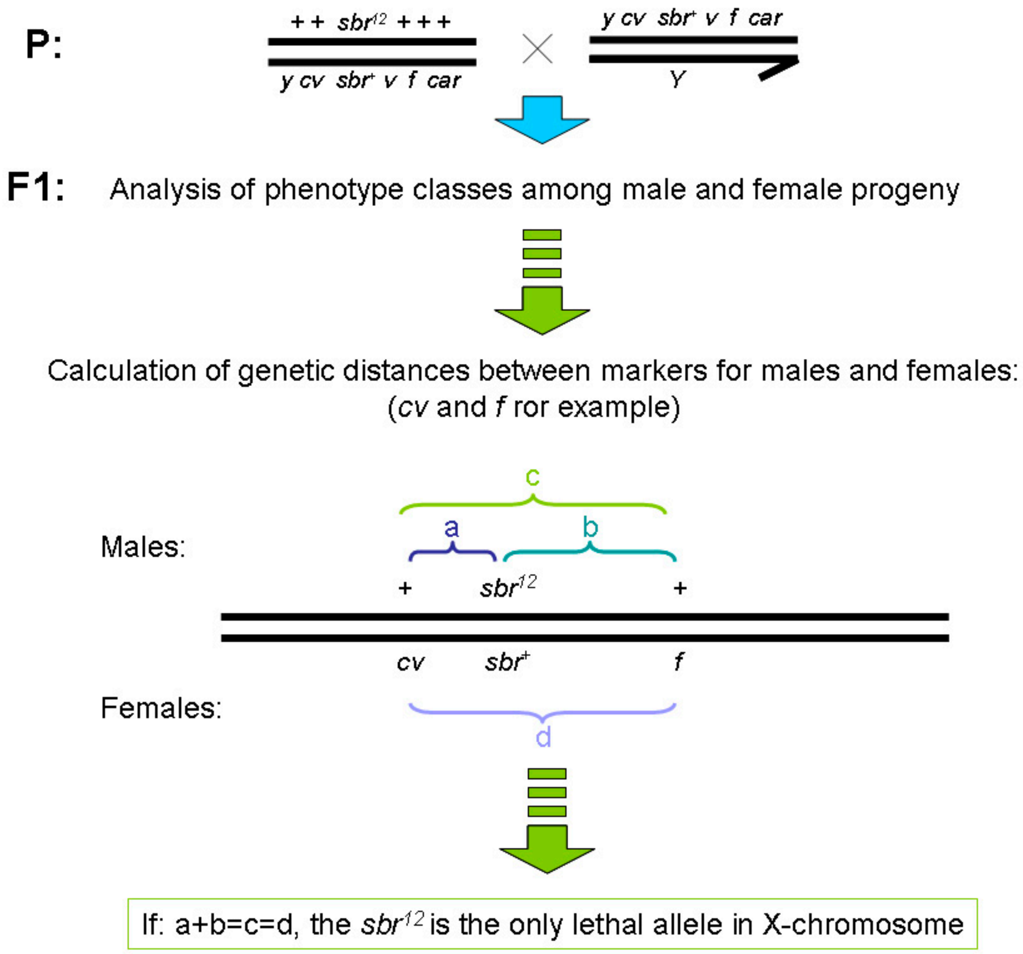

Supplement: Supplementary file 1 [file cells-10-01144-s001.zip › cells-1207878-supplementary/Appendix A ed.pdf]
