# Supplementary material for: The RNA-Binding Protein SBR (Dm NXF1) Is Required for the Constitution of Medulla Boundaries in Drosophila melanogaster Optic Lobes
Source: Cells. 2021 May 10;10(5):1144. doi: 10.3390/cells10051144 (PMC8151460; doi:10.3390/cells10051144)
Supplement: Supplementary file 1 [file cells-10-01144-s001.zip › cells-1207878-supplementary/suppl ed.pdf]

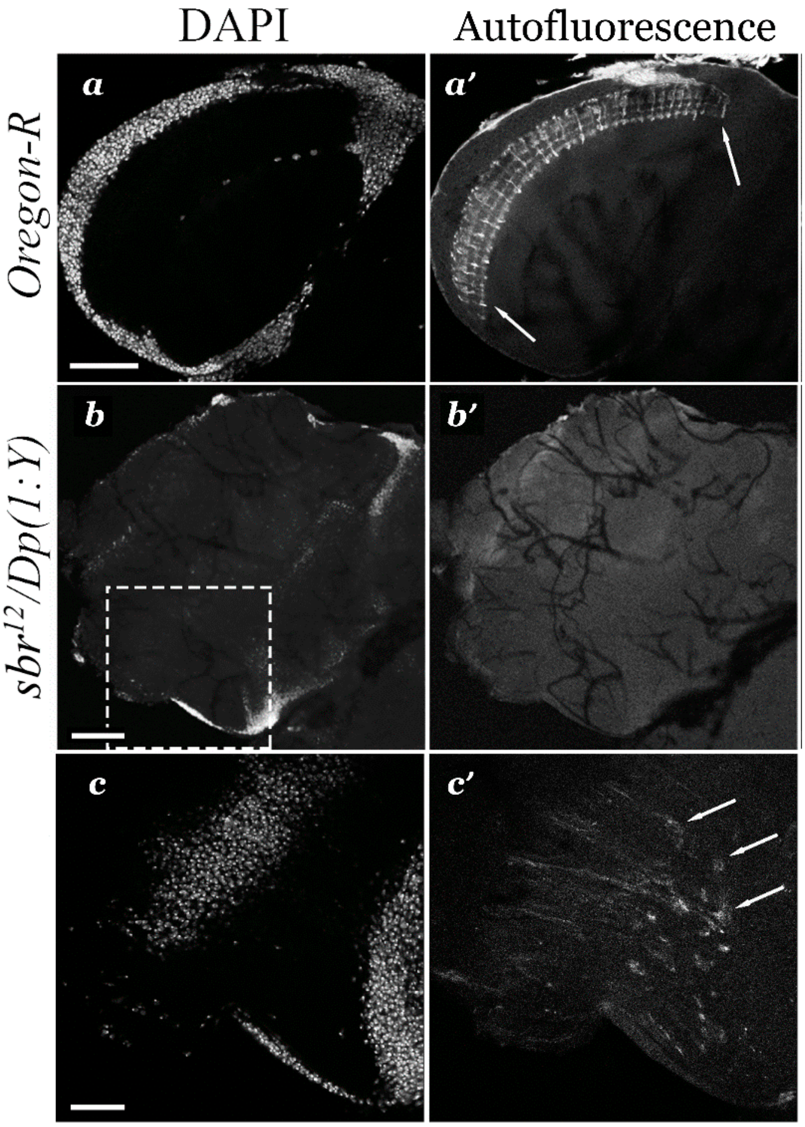

Figure S1. Axon terminals of the photoreceptors in the medulla of the optic lobe in adult males of different genotypes (*a, a´* – Oregon-R, *b, b´, c, c´* – *sbr^{12}/Dp(1;Y)y^+v^+*). Cell nuclei were stained with DAPI (*a, b, c*). Autofluorescence allows to detect photoreceptor axon terminals (*a´, c´*). Arrows indicate the R7-R8 axon terminals in the distal part of the medulla in Oregon-R (*a´*). In *sbr^{12}/Dp(1;Y)y^+v^+*, autofluorescence (*b´, c´*) is weaker, then in *Oregon-R*. The medulla structure is disrupted (*b, b´*). Axon terminals are not ordered and can be visualized by autofluorescence in the region marked on the top image (*b*) with higher magnification (*c´*). Scale bar: a, a′, b, b′ – 50 μm; c, c′ – 25 μm.

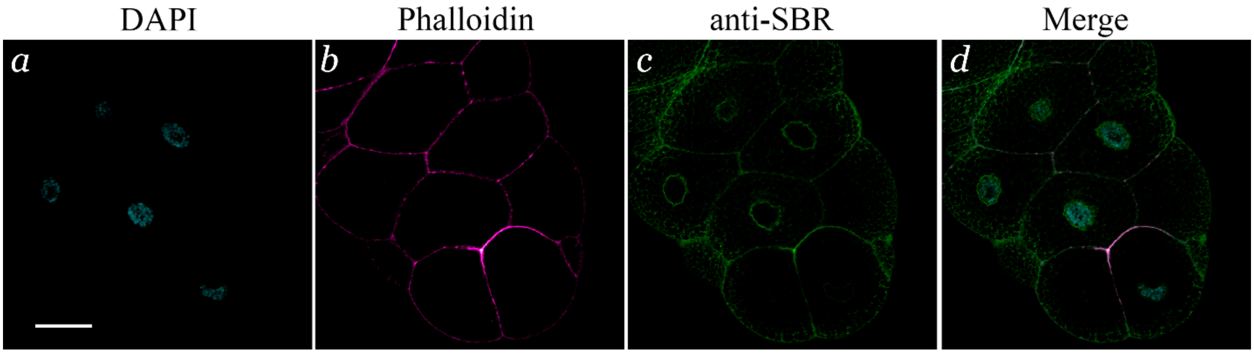

Figure S2. Localization of the SBR protein in cells of the salivary glands of *Drosophila*. The SBR protein is mark nuclear envelop and cellular membrane. a) DAPI (DNA stain); b) phalloidin (F-actin stain); c) anti-SBR; d) merge. Scale bar: 50 μm.
